# Disparities in Survival with Bystander CPR following Cardiopulmonary Arrest Based on Neighborhood Characteristics

**DOI:** 10.1155/2016/6983750

**Published:** 2016-06-09

**Authors:** Nina Thakkar Rivera, Shari L. Kumar, Rohit K. Bhandari, Sunil D. Kumar

**Affiliations:** ^1^Heart Center of Excellence, Broward Health Medical Center, 1600 South Andrews Avenue, Fort Lauderdale, FL 33316, USA; ^2^George Washington University, 2121 1st Street NW, Washington, DC 20052, USA; ^3^Department of Internal Medicine, Broward Health Medical Center, 1600 South Andrews Avenue, Fort Lauderdale, FL 33316, USA; ^4^Department of Pulmonary and Critical Care Medicine, Broward Health Medical Center, 1600 South Andrews Avenue, Fort Lauderdale, FL 33316, USA

## Abstract

The American Heart Association reports the annual incidence of out-of-hospital cardiopulmonary arrests (OHCA) is greater than 300,000 with a survival rate of 9.5%. Bystander cardiopulmonary resuscitation (CPR) saves one life for every 30, with a 10% decrease in survival associated with every minute of delay in CPR initiation. Bystander CPR and training vary widely by region. We conducted a retrospective study of 320 persons who suffered OHCA in South Florida over 25 months. Increased survival, overall and with bystander CPR, was seen with increasing income (*p* = 0.05), with a stronger disparity between low- and high-income neighborhoods (*p* = 0.01 and *p* = 0.03, resp.). Survival with bystander CPR was statistically greater in white- versus black-predominant neighborhoods (*p* = 0.04). Increased survival, overall and with bystander CPR, was seen with high- versus low-education neighborhoods (*p* = 0.03). Neighborhoods with more high school age persons displayed the lowest survival. We discovered a significant disparity in OHCA survival within neighborhoods of low-income, black-predominance, and low-education. Reduced survival was seen in neighborhoods with larger populations of high school students. This group is a potential target for training, and instruction can conceivably change survival outcomes in these neighborhoods, closing the gap, thus improving survival for all.

## 1. Introduction

According to the American Heart Association (AHA), in 2013 the incidence of out-of-hospital cardiopulmonary arrests (OHCA) was 359,400, of which the overall survival rate was 9.5% [1]. Of those arrests, 40.1% received bystander cardiopulmonary resuscitation (CPR) [1]. Among areas being surveyed by the Resuscitation Outcomes Consortium, there was a greater than 5-fold regional variation [2, 3]. In 2012, the AHA determined survival following OHCA in Detroit, Michigan, was as low as 0.2% as compared to 16% in Seattle, Washington [3, 4]. This vast geographical disparity is thought to be partly due to incidence and performance-quality of bystander CPR.

Observational studies proposed one life saved for every 30 persons that received bystander CPR [5]. Mortality translated not only to survival at hospital arrival, but also to survival at 30 days and one year after arrest [6]. Moreover, a 10% decrease in survival was associated with every minute of delay in initiating CPR [7]. Incidence of bystander CPR varies widely from 2 out of every 3 to one out of every 10 OHCA [2, 3, 8–10]. The least percentages of bystander CPR incidences have been described in rural, low-income, and minority-predominant neighborhoods [11–15]. Furthermore, areas with the largest rates of bystander CPR performance experienced higher rates of survival [8, 16].

In 2014, a nationwide study determined that CPR training was rare and differed extensively by location [17]. As expected, the lowest rates of CPR training occurred in neighborhoods that were rural, low-income, and minority-predominant [17]. Altogether, this is consistent with lower survival rates in rural, low-income, and minority-predominant neighborhoods, most likely due to lower incidence and poor performance-quality of bystander CPR, which is likely worsened by reduced CPR training.

Over the past 30 years, overall survival following OHCA has been consistently less than 10% [3, 5, 8, 9]. Consequently, the AHA has put forth a strong initiative to heighten bystander CPR in all areas by way of the Hands-Only*™* CPR campaign, which provides public service announcements, digital promotions, an animated instructional video, and a mobile CPR training tour [18, 19]. Additionally, the Centers for Disease Control in collaboration with the AHA and Emory University developed the Cardiac Arrest Registry to Enhance Survival to investigate the critical time between OHCA and hospital arrival [20].

To aid in the advancement efforts for improving survival following OHCA, more research is needed to define the areas of disparity. Identification of these areas will allow for direct-targeted training strategies to be designed and implemented. Therefore, we analyzed OHCA occurring over a 25-month period in a major metropolitan county in South Florida to assess the impact of neighborhood race, income, and education on bystander CPR and survival. Furthermore, we investigated the relationship between bystander CPR and survival with population characteristics of number of high school age persons. Finally, we propose a strategic plan to enhance bystander CPR incidence and performance-quality resulting in increased survival potential.

## 2. Methods

We conducted a retrospective study of persons who suffered OHCA in Broward County, Florida, with associated medical care at Broward Health Hospitals over a 25-month period. Of 320 patients with OHCA, 195 were excluded that did not meet the eligible criteria. “Noncardiac” causes of OHCA were excluded, such as trauma, drowning, overdose, asphyxia, and electrocution. As this study's interest was directly tied to performance of layperson bystander CPR, we excluded OHCA that occurred in a medical facility with on-site healthcare professionals (nursing homes, hospitals, medical clinics, and air ambulances), those witnessed by healthcare professionals, and those that occurred in a facility with basic life support-certified staff (airports and jails).

Using records from Broward County Fire Rescue and Broward Health Hospitals, the following variables were evaluated: location of OHCA (home versus street), zip code of OHCA, presence of a witness, performance of bystander CPR, initial cardiac rhythm, time until CPR, time until return of spontaneous circulation (ROSC), outcome (survival or death), and cause of death. The primary endpoint was designated as either ROSC following initial CPR immediately following OHCA or determination of death.

The Broward County Geographic Information Systems and United States Department of Commerce/United States Census Bureau information were employed to collect data on median income, racial breakdown, percentage of high school graduates, and number of persons 15 to 19 years of age per zip code. Annual income brackets were separated based on local economic trends in salaries and earnings and divided into three categories: less than $31,000 (low), $31,000 to $50,000 (intermediate), and greater than $50,000 (high). Neighborhoods were classified as white-predominant or black-predominant if greater than 80% of the population in that neighborhood was of that race or integrated if there was no predominance. Neighborhoods were separated into low- (less than 75%), intermediate- (75% to 89%), and high-education (greater than 89%) based on the percent of population with education of high school and above. Finally, neighborhoods were separated based on the number of high school age persons per zip code: less than 1,000 (low), 1,000 to 5,000 (intermediate), and greater than 5,000 (high).

Microsoft Excel® was used to conduct chi-square test analyses. *p* values determined statistical significance as less than or equal to 0.05. Institutional review board approval was obtained in advance to initiate this research study.

## 3. Results

### 3.1. Patient Demographics and Neighborhood Characteristics

Patient demographics and neighborhood characteristics are displayed (Table 1). All population sizes were statistically comparable amongst the variables discussed.

### 3.2. Variations in Survival by Neighborhood Income, Race, and Education Level

**Figure 1:**
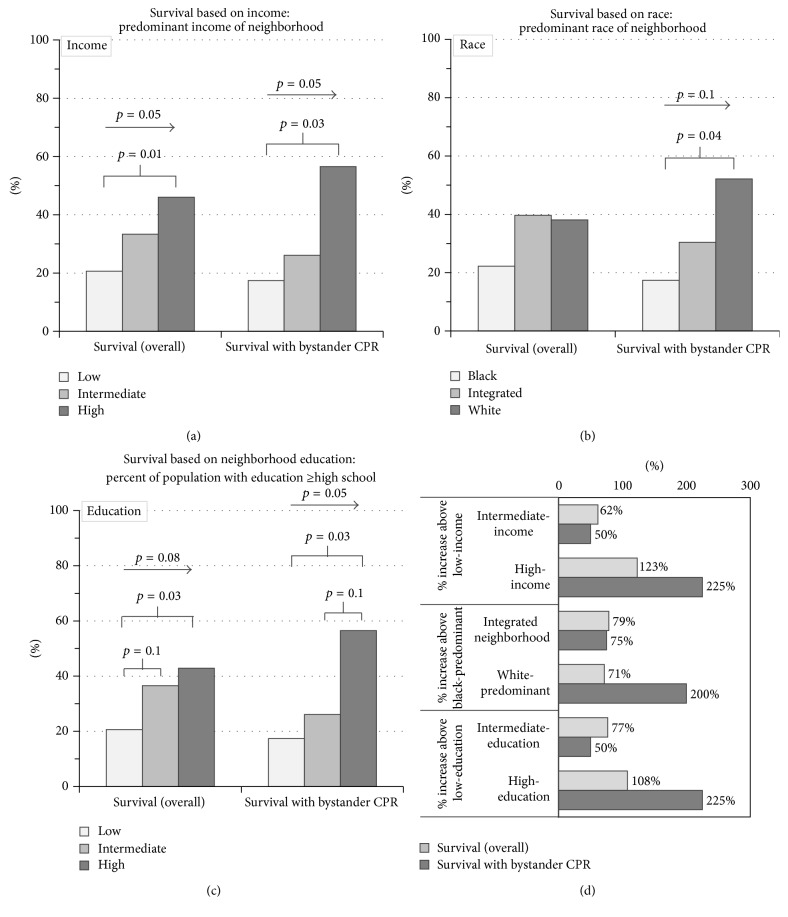
Survival based on income, race, and education level. Neighborhoods were separated by (a) income bracket, (b) race-predominance, and (c) education level. Overall survival (left) and survival with bystander CPR (right) are displayed. *p* values with arrows indicate trends across the 3 groups. *p* values with underlying brackets indicate differences between the two populations under each arm of the bracket. (d) Percent increased survival in intermediate-income and high-income versus low-income neighborhoods (upper half), integrated and white-predominant versus black-predominant neighborhoods (middle half), and high- and intermediate-education level versus low-education level (lower half) is shown.

Analysis of socioeconomic status in relation to OHCA revealed a statistically significant trend in both overall survival (*p* = 0.05) and survival with bystander CPR (*p* = 0.05), such that there was greater survival as the income bracket of the neighborhood increased (Figure 1(a)). The statistical significance was even greater when comparing low- to high-income neighborhoods with respect to overall survival (*p* = 0.01) and survival with bystander CPR (*p* = 0.03) (Figure 1(a)).

Quantification of the improved outcome of survival revealed more than two times greater (a 123% increase) rate of survival in high- versus low-income neighborhoods (Figure 1(d)), which was even more striking with bystander CPR, specifically more than three times greater (a 225% increase) (Figure 1(d)).

Although not statistically significant (*p* = 0.16), the number of bystander CPR times performed was nearly double in the high-income neighborhoods as compared to both the intermediate- and low-income brackets. It is likely that both reduced incidence and performance-quality of bystander CPR played a role in the disparity between the neighborhoods.

Race-based population analyses also demonstrated statistically significant trends, specifically in survival with bystander CPR between white-predominant and black-predominant neighborhoods (*p* = 0.04) (Figure 1(b)). There was a substantial trend of improved survival with bystander CPR transitioning from black-predominant to integrated to white-predominant neighborhoods (*p* = 0.1) (Figure 1(b)).

Quantification of differences in survival disclosed notable findings. Bystander CPR resulted in three times greater survival (a 200% increase) in white-predominant versus black-predominant neighborhoods (Figure 1(d)). Survival with bystander CPR nearly doubled (a 75% increase) in integrated versus black-predominant neighborhoods (Figure 1(d)). A similar increase (71%) in survival with bystander CPR was seen in integrated versus white-predominant neighborhoods. The disparity was less pronounced in overall survival with near doubling of overall survival (a 71% increase) in white-predominant versus black-predominant neighborhoods (Figure 1(d)). There was no statistical difference in incidence of bystander CPR, which introduces the caveat that performance-quality of bystander CPR was likely diminished in black-predominant neighborhoods.

Neighborhoods categorized by education level also provided analogous results. Analysis of overall survival noted a substantial trend such that the higher the percentage of education persons within a neighborhood, the greater the rates of overall survival (*p* = 0.08) (Figure 1(c)). There was a statistically significant increased rate of both overall survival (*p* = 0.03) and survival with bystander CPR (*p* = 0.03) when comparing high- versus low-education neighborhoods (Figure 1(c)). Further, there was a statistically significant trend in survival with bystander CPR directly proportional to having a greater percentage of the neighborhood population with high school education and above (*p* = 0.05) (Figure 1(c)).

Quantification revealed survival resulting from bystander CPR was more than three times greater (a 225% increase) in high-education neighborhoods (greater than 89%) as compared to low-education neighborhoods (less than 75%) (Figure 1(d)). Although not statistically significant (*p* = 0.16), the number of bystander CPR times performed was nearly double in neighborhoods with the highest percentage of educated persons. Altogether, this suggests that both reduced incidence of initiating bystander CPR and performance-quality of bystander CPR contribute to the disparity of survival in neighborhoods with a lower percentage of educated persons.

### 3.3. Survival in Association with Time and Effect of Bystander CPR on Overall Survival

**Figure 2:**
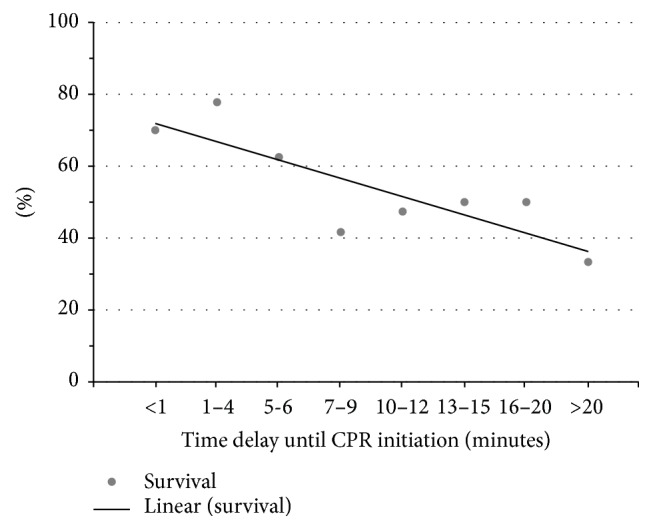
Effect of time delay in initiation of CPR on survival. Patients were evaluated based on time delay in CPR initiation. A trend line is displayed with an equation of *y* = −0.05*x* + 0.77 and a coefficient of determination (*R*-squared) of 0.7, indicating a favorable goodness of fit in correlation of the improved survival with reduced time delays in CPR initiation.

Previous studies published by the AHA indicated a relationship between delay in the initiation of CPR and survival and thereby the crucial role of bystander CPR [17]. Therefore, the total population was analyzed with respect to time delay until CPR initiation. With each one-minute incremental delay in initiating CPR, there was a 7.2% decrease in survival (Figure 2). The coefficient of determination was 0.7 indicating a goodness of fit in correlation.

**Figure 3:**
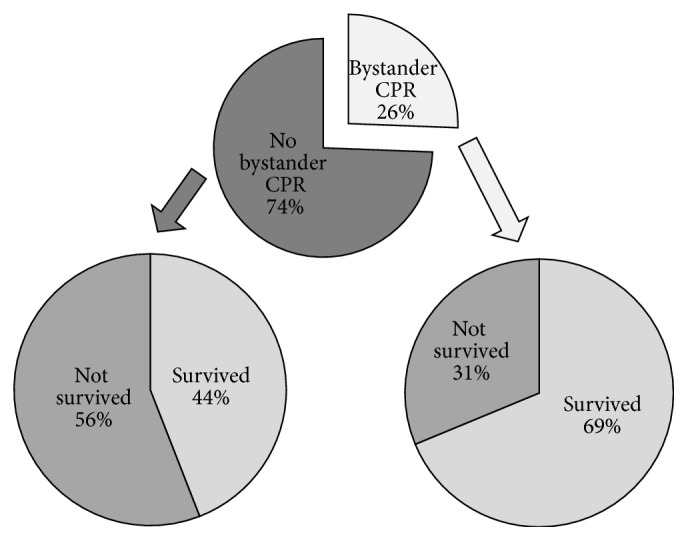
Effect of bystander CPR on survival. Patients were divided into those that received bystander CPR and those that did not (top pie chart). Each group was further evaluated for outcome of survival in association with having received bystander CPR (bottom right pie chart) and not having received bystander CPR (bottom left pie chart).

The total population was further evaluated for overall incidence of bystander CPR and its impact on survival (Figure 3). Of the 125 OHCA, 86 were witnessed, 63 survived, and 34 received bystander CPR. The overall survival was 50.4%. When bystander CPR was excluded, survival dropped to 44%, as compared to 69% with bystander CPR (Figure 3), further indicating the grave importance of bystander CPR in survival outcome following OHCA.

### 3.4. Variations in Survival by High School Population

**Figure 4:**
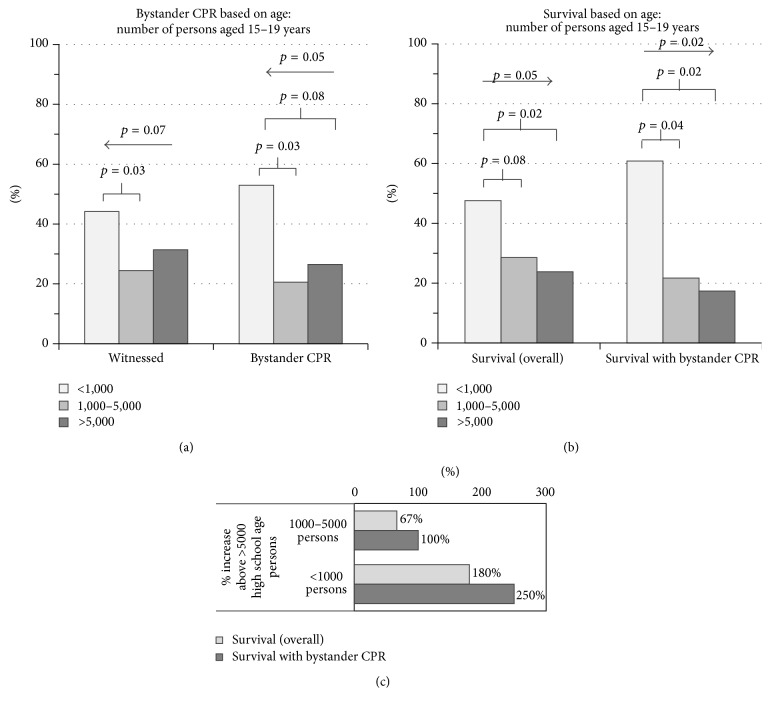
Bystander CPR based on number of high school age persons. (a) Neighborhoods were separated by number of high school age persons. Percentages of witnessed cardiopulmonary arrests (left) and bystander CPR (right) are displayed. (b) Overall survival (left) and survival with bystander CPR (right) are displayed. *p* values with arrows indicate trends across the 3 groups. *p* values with underlying brackets indicate differences between the two populations under each arm of the bracket. (c) Percent increased survival in low number and intermediate number of high school age persons versus high number of high school age persons.

As a potential target group of training, survival was also examined with relation to high school age populations (15 to 19 years of age). In neighborhoods with low high school age persons, there were more witnessed OHCA and more events of performed bystander CPR (Figure 4(a)). There was a substantial trend revealing more witnessed OHCA (*p* = 0.07) and more events of bystander CPR (*p* = 0.05) inversely proportional to the number of high school age persons (Figure 4(a)). A statistically significant greater number of witnessed OHCA (*p* = 0.03) and performed bystander CPR (*p* = 0.03) in the neighborhoods with low versus intermediate high school age persons (Figure 4(a)).

Overall survival and survival with bystander CPR followed similar trends. There was a statistically significant trend revealing greater overall survival (*p* = 0.05) and survival with bystander CPR (*p* = 0.02) inversely proportional to the number of high school age persons (Figure 4(b)). Further, there were statistically significant greater overall survival (*p* = 0.02) and survival with bystander CPR (*p* = 0.02) in the neighborhoods with low verses high school age persons (Figure 4(b)). Although not statistically significant with overall survival (*p* = 0.08), there was a statistically significant higher rate of survival with bystander CPR (*p* = 0.04) in neighborhoods with low versus intermediate high school age persons (Figure 4(b)).

Quantification of these differences in survival provided similar correlations. There was more than three times greater (a 250% increase) survival with bystander CPR in neighborhoods with low versus high school age persons (Figure 4(c)). The trend was also significant, specifically nearly tripling (a 180% increase) of survival with bystander CPR in neighborhoods with low versus intermediate high school age persons (Figure 4(c)). Altogether, these data reveal that there is no statistical difference in witnessing of OHCA in neighborhoods with a larger number of high school age persons, but there is a statistically significant reduced incidence of bystander CPR, overall survival, and survival with bystander CPR.

## 4. Discussion

Our observational, retrospective study revealed a direct relationship between neighborhood income, race, and education level with survival from OHCA, both overall and with bystander CPR. A direct correlation of increased survival associated with increased neighborhood income was found, most evident in comparison of the low- and high-income brackets. Similar associations were found with respect to neighborhood race-predominance, specifically the lowest survival with bystander CPR seen in black-predominant neighborhoods. Parallel results were seen in populations distinguished by percent educated, such that neighborhoods with more educated persons had greater survival, both overall and with bystander CPR.

The AHA determined a 10% increase in mortality with each one-minute delay in initiation of CPR [7]. Our study reports similar results with a mortality increase of 7.2%. This further underscores the importance of bystander CPR. In our study, there was a significant improvement in survival when comparing outcomes with and without bystander CPR. This data is also well established in the literature [5, 6, 8, 16]. However, the disparity in survival varies vastly by region [2, 3, 7–10, 17] indicating a role for both incidence and performance quality of CPR.

Our study clearly defines the decreased incidence in bystander CPR performance in neighborhoods with greater high school age persons despite comparable incidence in witnessed OHCA. Further evidently, there is increased mortality in these areas with greater high school age persons. Therefore, the population category of high school students is a strong, potential target group for future training.

Recent focus has been on evaluating this population subset for CPR training. In 2003, the International Liaison Committee on Resuscitation made recommendations for CPR training to become included into school curriculums [21]. In 2004, the AHA initiated a movement to train teachers in CPR, such that they may extend training to students for medical emergency response on school grounds [22]. By 2010, 36 states encouraged CPR training incorporation into school curricula; however execution was inconsistent [23]. For that purpose, the AHA released a statement recommending CPR training and understanding of automated external defibrillation (AED) use become required in high schools [23].

Multiple studies have shown that the rate of bystander CPR performance was significantly higher when persons were trained [10, 24]. The education system offers an excellent population pool for establishment and integration of CPR training. Numerous studies have evaluated competency of persons following training in different age brackets [25–32]. These studies indicated that retention of training and subsequent adeptness with CPR performance was greatly reduced in 10 to 12 year olds [26, 27, 32]. However in a slightly older age bracket of 12 to 14 year olds, significant proficiency with CPR skills was demonstrated 4 weeks after training [29]. In another study, 13 to 14 year olds performed chest compressions on the level of adults [28]. In evaluation of CPR training efficacy on short-term and longer-term performance-quality among high school students, four-month follow-up demonstrated remarkable 99% still proficient with chest compressions [31]. Interestingly, nearly 100% of those trained admitted feeling more confident about performing CPR in an emergency medical situation, as compared to 27% prior to training [31].

Questions have been raised as to whether high school students would be a practical group to be trained. Although the majority of OHCA occur in the older population and there is an extremely low incidence of OHCA that occur in schools [33], upwards of two-thirds occur at home [34] and another large percentage occurs in local areas that high school students frequent, such as shopping malls and sporting venues [35]. In a recent study, one in 5 students was exposed to an emergency medical event, but only one out of 4 in those situations actually attempted to perform CPR [36]. Cost-analysis of implementing CPR and AED training in high schools determined the cost per student was around 9 dollars [36]. High schools are both localized and organized. Establishing CPR training as a graduation-requirement aids in easy implementation. Altogether, this indicates that training high school students in CPR is not only realistic in terms of location and exposure but also practical with regard to efficacy and cost-effectiveness.

### 4.1. Limitations

There are important limitations within our study. The population size is small and localized lending for the possibility of sampling bias. Being retrospective, it is problematic to rely on others for accurate records. There is no centralized database from the county EMS. Altogether, this can introduce information bias into the study. Finally, argument can also be made that there are greater morbidity and associated mortality in persons of minority race, low-income, and low education, providing room for a confounding variable.

## 5. Conclusions

We discovered a significant disparity in survival following OHCA within neighborhoods of low-income, black-predominance, and poor education. Neighborhoods with reduced survival had a large population of high school students, making them a potential target for future training and allowing for early intervention. There is more power in training high school age persons in one location and this instruction can conceivably change survival outcomes following OHCA in these unfortunate neighborhoods. By enhancing both incidence and performance-quality of bystander CPR, training of local high school populations within each neighborhood can hopefully close the gap between races, income-status, and education level, resulting in improved survival for all.

## Figures and Tables

**(a):** 

Gender			
Male	20	22	31
Female	22	16	14

Location			
Home	33	19	22
Street	9	19	23

Initial rhythm			
Asystole	22	18	19
PEA	11	8	10
VF	8	10	15
Pulseless VT	1	0	0
Unknown	0	2	1

**(b):** 

Gender			
Male	21	25	27
Female	22	16	14

Location			
Home	33	21	20
Street	10	20	21

Initial rhythm			
Asystole	23	19	17
PEA	11	9	9
VF	8	12	13
Pulseless VT	1	0	0
Unknown	0	1	2

**(c):** 

Gender			
Male	32	20	21
Female	17	12	23

Location			
Home	21	20	33
Street	28	12	11

Initial rhythm			
Asystole	20	17	22
PEA	9	8	12
VF	18	6	0
Pulseless VT	0	0	1
Unknown	2	1	0

**(d):** 

Gender			
Male	20	23	30
Female	22	16	14

Location			
Home	33	19	22
Street	9	20	22

Initial rhythm			
Asystole	22	18	19
PEA	11	9	9
VF	9	11	14
Pulseless VT	1	0	0
Unknown	0	1	2

## Abbreviations

AED: Automated external defibrillation
AHA: American Heart Association
CPR: Cardiopulmonary resuscitation
EMS: Emergency Medical Services
OHCA: Out-of-hospital cardiopulmonary arrest
ROSC: Return of spontaneous circulation.
